# The effects of Malaysian propolis and Brazilian red propolis on connective tissue fibroblasts in the wound healing process

**DOI:** 10.1186/s12906-015-0814-1

**Published:** 2015-08-25

**Authors:** Ann Jacob, Abhishek Parolia, Allan Pau, Fabian Davamani Amalraj

**Affiliations:** School of Medicine, International Medical University, Kuala Lumpur, Malaysia; School of Dentistry, International Medical University, Kuala Lumpur, Malaysia; School of Human Biology, International Medical University, Kuala Lumpur, Malaysia

**Keywords:** Wound healing, Propolis, Fibroblast

## Abstract

**Background:**

To evaluate and compare the effects of ethanolic extracts of Malaysian propolis and Brazilian red propolis at different concentrations on the migration and proliferation of fibroblast cells.

**Methods:**

Malaysian and Brazilian red propolis crude samples were extracted using ethanol. Their wound healing effects were tested *in vitro* on the normal human fibroblast cell line CRL-7522. Cell migration and proliferation assays were carried out using propolis concentrations of 1, 10, 100, 250, 500 and 1000 μg/mL. The data were analyzed using one-way ANOVA and post hoc Bonferroni tests (α = 0.05).

**Results:**

Malaysian and Brazilian red propolis followed a concentration-dependent increasing and decreasing trend. Malaysian propolis showed the fastest migration rate at 250 μg/mL which was statistically significant (*p* < 0.05) and maximum proliferation at 500 μg/mL with no significant difference (*p* > 0.05) compared to control. Brazilian red propolis showed a slight increase in migration and proliferation at 10 and 100 μg/mL, respectively with no significant difference (*p* > 0.05) compared to control, while concentrations above these conferred inhibitory effects.

**Conclusion:**

Malaysian and Brazilian red propolis show potential to assist in wound healing, depending on their concentration.

## Background

Wound healing is an intricate and continuous process of tissue repair that begins immediately following an injury. The physiological process consists of a series of cascading, yet overlapping stages that can be divided into three main phases: inflammatory, proliferative and remodeling, involving various cells such as keratinocytes, fibroblasts and endothelial cells [1]. The most important cells among these are fibroblasts, responsible for initiating angiogenesis, epithelialization and collagen formation [1]. In terms of dentistry, the maintenance of a healthy and functional pulp-dentine complex is essential and any injury to the pulp tissue may affect the vitality of the tooth structure. Like other connective tissues, pulp tissue has the potential to heal, and fibroblast cells play a key role in the healing process [2]. This process is vital in our everyday lives and consequently, the effect of various substances on the wound healing process is the subject of continuing translational research. Although many studies have been conducted to evaluate the healing potential of various therapeutic agents, there is still a scarcity of scientific reports on agents that can facilitate the process of wound healing at a cellular level. In recent years, there has been increasing interest in evidence-based complementary and alternative medicine, thus resulting in multifarious studies on the use of natural products in wound healing. Propolis, a natural product, has been one part of that extensive research. Propolis is a substance collected by honey bees from a plant’s buds and exudates and consists mainly of resins, balsams, beeswax, essential oils, pollen and other organic compounds [3]. Propolis, due to its antibacterial, antifungal, antiviral, antitumour, anti-oxidative, immunomodulatory, anti-diabetic, anti-ulcer and healing properties, has been extensively used in the health industry worldwide [4–6]. Recently, scientists have investigated these medicinal properties by examining the chemical compositions of various types of propolis and the biological actions of those compounds [7]. The chemical composition of propolis varies with the geographic location and type of plant that it is collected from. Brazilian propolis is classified into 12 types according to physicochemical properties and geographic locations; however, only three types are identified related to the botanical origin. A thirteenth type of Brazilian propolis has been recently identified in the state of Alagoas, named Brazilian red propolis due to its red colour [7]. This red propolis has been discovered in beehives along the shorelines of the northeastern region of Brazil, and *Apis mellifera* bees have been found to gather these reddish exudates from the surface of *Dalbergia ecastophyllum (L.) Taub* [7, 8]. Brazilian red propolis was found to be rich in isoflavanoids, a chemical compound that has been researched extensively and is believed to be responsible for the majority of biological properties inherent in propolis [8–10]. Studies have suggested that propolis could confer either a stimulatory or an inhibitory effect on the migration and proliferation of cells involved in healing, depending on the cell type, type of propolis and possibly its concentration [11, 12]. Although many of the biological properties of propolis, such as its antimicrobial and anti-oxidative activities, may aid in the wound healing process, they do not directly relate to the proliferative phase of wound healing. To date, no scientific study has been carried out on the use of Malaysian propolis in wound healing. Therefore, given the limited studies carried out on the effects of Brazilian red propolis on the proliferative phase of wound healing and the lack of scientific study on Malaysian propolis, this present study was aimed to evaluate and compare the effects of ethanolic extracts of Malaysian propolis and Brazilian red propolis at different concentrations (1, 10, 100, 250, 500 and 1000 μg/mL) on the migration and proliferation of connective tissue fibroblast cells, and to determine the optimum association curve between time and migration of the fibroblast cells at the different concentrations of Malaysian propolis and Brazilian red propolis.

## Methods

### Propolis sources

Two types of propolis were tested in this study: Malaysian and Brazilian red propolis. The Malaysian propolis sample was supplied by Econest Enterprise Sdn. Bhd, Malaysia. The propolis was obtained in July, from a fruit farm in Gurun, Kedah and is known to be collected by the stingless honey bee *Trigona spp.* The Brazilian red propolis sample was collected in July, from the Number One apiary on Camarões farm in Barra de Santo Antônio city, Alagoas, Brazil, with geographical coordinates of South latitude: 9° 24′.58″, West latitude: 35° 30′.33″. It is collected by the European honey bee *Apis mellifera sp.*

### Extraction process

The propolis was cut into small pieces. 40 g were weighed using a weighing balance (Pyrometro, Malaysia) and divided equally into 4 flasks with 10 g each. Then in the flask, propolis was mixed with 80 % ethanol at a ratio of 1 g: 10 mL and mixtures were shaken (Certomat Model S II, Sartorius, Goettingen, Germany) at 200 rpm for 24 h at 60 °C and centrifuged (Eppendorf Model 5810 R, Hamburg, Germany) at 3000 rpm for 15 min at 10 °C. Then the supernatant was filtered using paper filter. The low temperature was used to allow the wax to harden and be separated from the supernatant. The filtrate was evaporated using a rotary evaporator (Buchi Model R-215, Flawil, Switzerland) at the set pressure and temperature for ethanol solvent (175 mBar, 52 °C) at 95 rpm for 30 min. The remaining unevaporated filtrate (approximately 60 mL) was collected in a beaker and left to completely evaporate at room temperature in a fume hood (Erla, Captair Toxicap 1200 NU/ASP, Belgium, USA). Following complete evaporation, the result was a raw sticky semi-solid ethanol extract of propolis (EEP). Each gram of EEP was dissolved in 2 mL of dimethyl sulphoxide (DMSO) and mixed vigorously in a vortex mixer (Stuart Model SA8, Bibby Scientific, Staffordshire, UK).

### Cell culture

Human fibroblast cell line Hs 792(C) M (ATCC® CRL-7522™), obtained from American Type Cell Culture (ATCC, Manassas, USA), were maintained according to the protocol prescribed by ATCC.

After thawing, the cells were cultured in humidified incubators at 5 % (volume fraction) CO_2_, 37 °C and 70 % relative humidity in Dulbecco’s Modified Eagle’s Medium (DMEM) (Gibco, Oklahoma, USA) supplemented with 10 % Foetal Bovine Serum (FBS) (Gibco, Oklahoma, USA) and 1 % penicillin-streptomycin (Gibco, Oklahoma, USA). The cell suspension was spun at 1500 rpm for 3 min in a centrifuge (Eppendorf Model 5702, Hamburg, Germany), transferred to a 60 mm^2^ culture plate containing the complete growth medium and placed in an incubator (RS Biotech Model Galaxy S, Scotland, UK) under same conditions, to allow the cells to adhere to the bottom of the plate and grow.

The cell adherent layer was washed out with 5 mL of Phosphate Buffer Saline (PBS) (Gibco, Oklahoma, USA) and then 1 mL of 0.5 % trypsin (Gibco, Oklahoma, USA) was added to the plate. The cells were incubated for 1 min at 37 °C. The cell suspension was then transferred to a 15 mL centrifuge tube containing 5 mL of culture medium and centrifuged at 1500 rpm for 3 min. The supernatant was discarded and the cell pellet was gently re-suspended in 1 mL of fresh medium. The cell suspension was transferred to a 30 mm^2 ^culture plate for subculturing purposes and placed in the humidified incubator under the same conditions. Within three days approximately 85 % confluency was reached.

### Preparation of treatment concentrations

The concentration of the EEP stock was 0.5 g/mL. Prior to carrying out an experiment, a reference concentration of 5 mg/mL was made from the EEP stock (0.5 g/mL). This was performed by using a portion of the EEP stock and diluting it to 1 % by addition of complete culture medium. The mixture was vortexed (FineVortex, FINEPCR, Korea) until the extract was completely dissolved in the medium. Then serial dilutions were performed from the 5 mg/mL into the various concentrations being tested (1000, 500, 250, 100, 10, 1 μg/mL). Each concentration was added with medium and made up to the required volume and stored in 1.5 mL microcentrifuge tubes and vortexed vigorously.

### Cell migration assay

CRL-7522 cells were seeded in 24-well plates (Nunc, Denmark) with 5 × 10^4^ cells/well and was incubated for two to three days until approximately 90 % confluency was reached. The medium was replaced every three days. A scratch wound was made on the monolayers in each well using a 10–200 μL pipette-tip. The plates were marked using a marker pen at the midpoint of each row of wells, on both sides, so as to ensure the scratches were made in the same area in each well. The medium was removed and replaced with 400 μL of the different treatments (Malaysian propolis and Brazilian red propolis), the negative control (plain DMEM) and the positive control (DMEM with 10 μL/mL of DMSO). In order to dissolve the propolis extracts in the culture medium, a maximum of 10 μL/mL of DMSO was used to obtain the highest propolis treatment concentration. Thus we have used that volume of DMSO as the positive control to test if DMSO had a negative effect on cell migration and proliferation. Here, the negative control will be referred to as control and the positive control will be referred to as DMSO control.

The test with the Malaysian propolis and the test with the Brazilian red propolis were carried out separately at concentrations of 1, 10, 100, 250, 500, 1000 μg/mL, and the controls. Each treatment, including the controls, was tested in triplicates. Images were taken in three fields by an inverted light microscope (Motic Model AE31, USA) at 4× magnification immediately after wounding (0 h), after 12 h, 24 h, and then at 6-hour intervals until complete closure of a wound. The migration of the cells were calculated by measuring the wound gap distance at fixed points in each photo, using Image J software (National Institutes of Health, USA). The rate of migration at each point was calculated using the formula below:

$$ \mathrm{Rate}\ \mathrm{of}\ \mathrm{migration}=\frac{\mathrm{Gap}\ \mathrm{distance}\ \mathrm{at}\ \mathrm{that}\ \mathrm{time}\ \mathrm{point}\hbox{--} \mathrm{Gap}\ \mathrm{distance}\ \mathrm{at}\ 0\ \mathrm{hour}}{\mathrm{Time}\ \mathrm{interval}\ \mathrm{from}\ 0\ \mathrm{hour}} $$

### Cell proliferation assay

CRL-7522 cells were seeded into 24-well plates at 1 × 10^4^ cells/well and the wells were added with 400 μL of either the different treatments or the controls. Each treatment, including the controls, was tested in triplicates. The plates were incubated for 24 h and 48 h. At each time point, the cells were removed from the wells using the subculture method as previously stated, and the cell pellets were re-suspended in 200 μL of PBS in 1.5 mL microcentrifuge tubes. The cells were counted using the trypan blue exclusion test using a Neubauer haemocytometer.

### Statistical analysis

Statistical analysis for testing of significance was carried out using one-way ANOVA, and post hoc Bonferroni tests were done where appropriate. A significance level of 0.05 was set, and the analysis was performed using SPSS Version 18.0 software (IBM SPSS Inc., Chicago, Illinois, USA).

## Results

### Cell migration assay

**Fig. 1:**
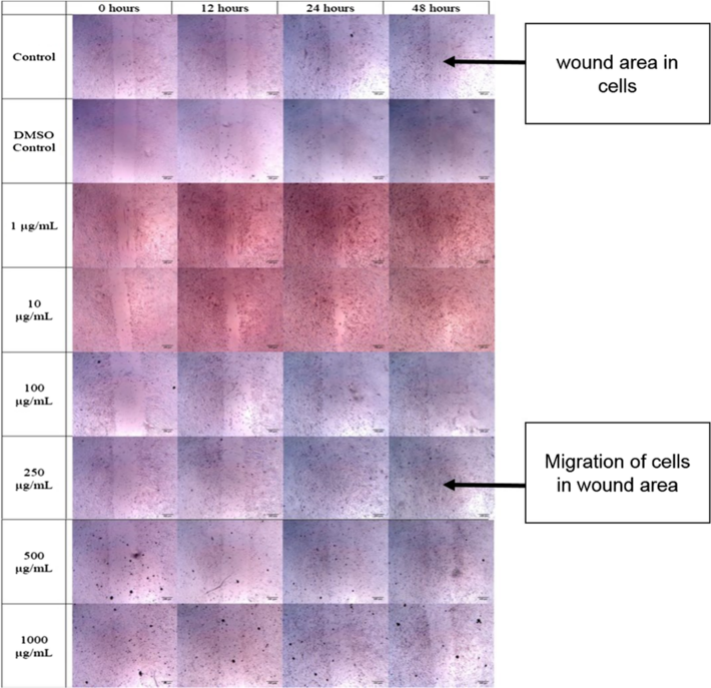
Microscopic images of wound areas in cells treated with different concentrations of Malaysian propolis at various time points. The experiment was carried out in triplicates. Images were taken at 4× magnification of the objective lens and the scale bars indicate a distance of 100 μm

Malaysian propolis at concentrations of 1, 10 and 250 μg/mL indicated visually faster wound closure compared to the control, while the other concentrations appeared visually similar to the control. The ability of cells to migrate into wounds in the presence of DMSO was similar to control at 12 h, but at 24 and 48 h the migration rate was slower than all other groups (Fig. 1).

**Fig. 2:**
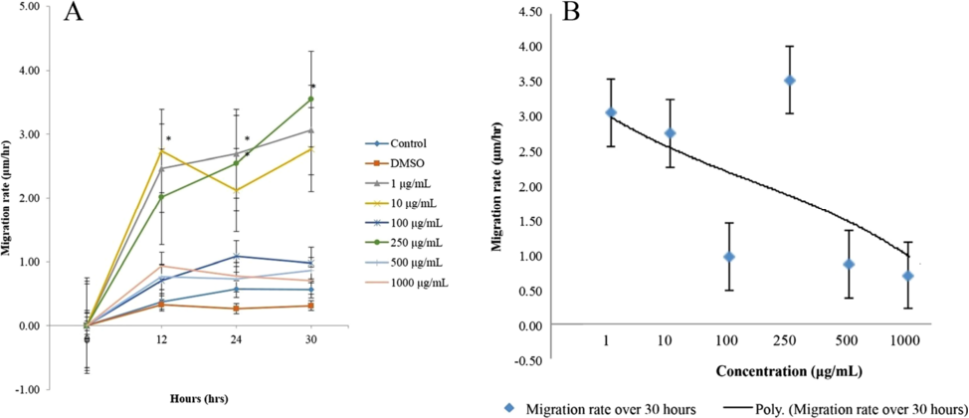
Migration rate of cells treated with Malaysian propolis. **a** Line graph showing migration rates of treated and untreated cells using different concentrations of Malaysian propolis at various time points. Values are expressed as mean migration rate ± standard error as indicated by the error bars. Significance: *p-value < 0.05 treatment vs control. **b** Polynomial trend line showing the concentration-dependent trend of the migration rate of cells treated with Malaysian propolis over 30 h. Values are expressed as mean migration rate ± standard error as indicated by the error bars

The concentrations from 1 to 1000 μg/mL revealed a higher migration rate compared to control throughout the entire assay duration (Fig. 2a). In the first 12 h, 10 μg/mL showed a significantly higher migration rate than control (*p* = 0.019). Subsequently, the 1 μg/mL (*p* = 0.009) and 250 μg/mL (*p* = 0.011) concentration migration rates became significantly faster than that of the control at 24 h. Notwithstanding, over the period of 30 h, only the 250 μg/mL concentration had a statistically significant migration rate higher than control (*p* = 0.021). Overall, there appeared to be a correlation between an increasing rate of migration in the treated cells and an increasing treatment concentration up to 250 μg/mL, from which point an inverse effect was observed with increasing treatment concentrations (Fig. 2b). The migration rate of cells treated with 100 μg/mL however did not coincide with the concentration-dependent increasing and decreasing trend shown by Malaysian propolis. DMSO group shows the lowest migration rate.

**Fig. 3:**
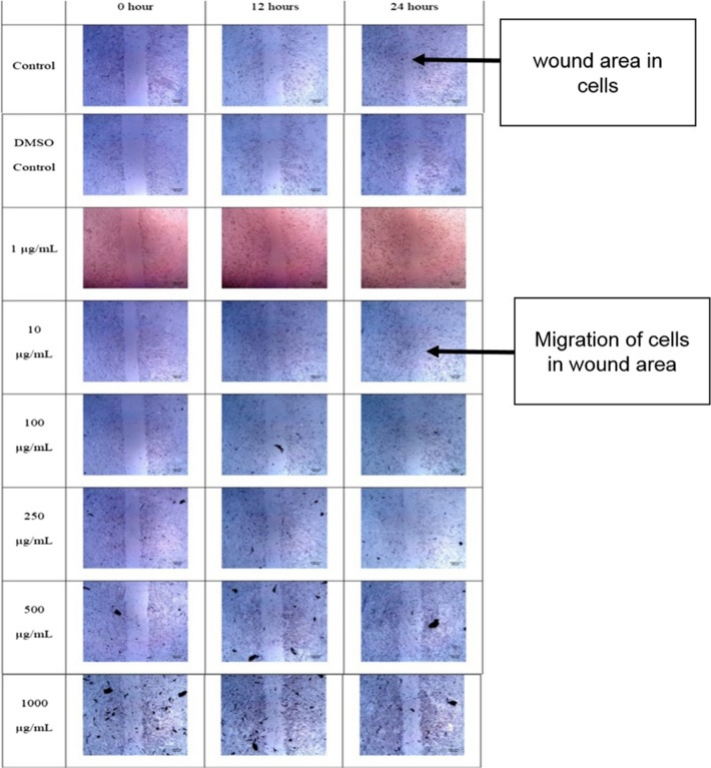
Microscopic images of wound areas in cells treated with different concentrations of Brazilian red propolis at various time points. The experiment was carried out in triplicates. Images were taken at 4× magnification of the objective lens and the scale bars indicate a distance of 100 μm

In the experiment using Brazilian red propolis, only the 10 μg/mL concentration indicated visually faster wound closures, compared to control. The 1 μg/mL concentration and DMSO control appear to indicate a similar migration rate to that of the control, while the other concentrations appear to visually show slower migration than the control (Fig. 3).

**Fig. 4:**
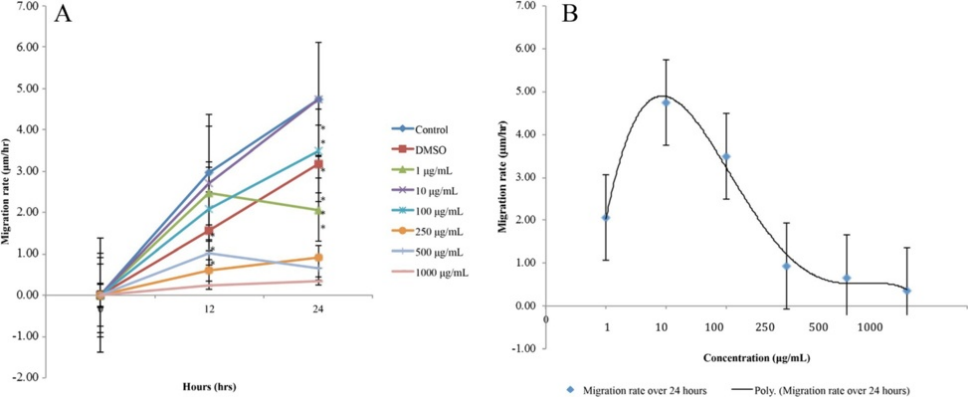
Migration rates of cells treated with Brazilian red propolis. **a** Line graph showing migration rates of treated and untreated cells using different concentrations of Brazilian red propolis at various time points. Values are expressed as mean migration rate ± standard error as indicated by the error bars. Significance: *p-value < 0.05 treatment vs control. **b** Polynomial trend line showing the concentration-dependent trend of the migration rate of cells treated with Brazilian red propolis over 24 h. Values are expressed as mean migration rate ± standard error as indicated by the error bars

In the first 12 h, the control showed the fastest migration rate compared to all treatment concentrations (Fig. 4a). However, only the 250 μg/mL (*p* = 0.001), 500 μg/mL (*p* = 0.01) and 1000 μg/mL (*p* = 0.000) concentrations showed a statistically slower migration rate than control. Over the 24–hour period, all treatment concentrations had a statistically significant slower average migration rate compared to that of the control (*p* < 0.05), with the exception of the 10 μg/mL concentration. The cells treated with 10 μg/mL concentration indicated a slightly faster rate of migration than the control, though not statistically significant (*p* > 0.05). Brazilian red propolis appeared to follow a concentration-dependent trend in its effect on fibroblast cell migration over the 24-hour period. The migration rate increased with an increase in concentration from 1 to 10 μg/mL, and then showed an inverse correlation as the migration rate declined as the concentration increased from 10 μg/mL (Fig. 4b). At 12 h cell migration rate in the presence of DMSO was higher than Brazilian red propolis at 250, 500 and 1000 μg/mL concentrations that indicates a potential cytotoxicity of Brazilian propolis more than 250 μg/mL concentrations (Fig. 4a).

### Cell proliferation assay

**Fig. 5:**
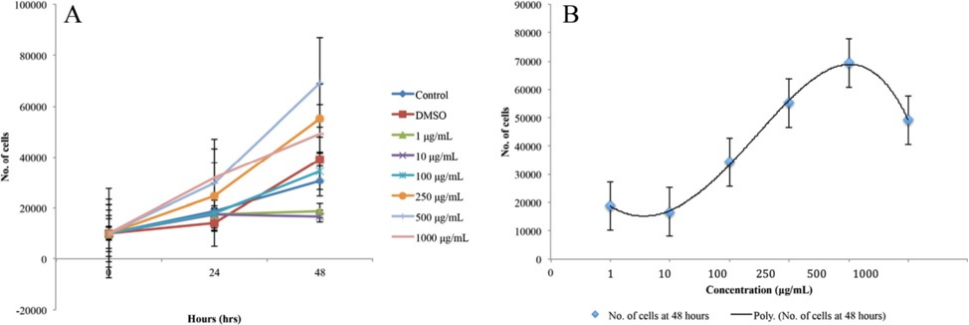
Proliferation of cells treated with Malaysian propolis. **a** Line graph showing proliferation of treated and untreated cells using different concentrations of Malaysian propolis at various time points. Values are expressed as mean number of cells ± standard error as indicated by the error bars. There was no significant difference between the mean number of cells of the control and treatments. **b** Polynomial trend line showing the concentration-dependent trend of the proliferation of cells treated with Malaysian propolis at 48 h. Values are expressed as mean number of cells ± standard error as indicated by the error bars

In the experiment using Malaysian propolis, the 500 μg/mL concentration showed the highest increase in cell count after 48 h. This was followed by 250 and 1000 μg/mL concentrations respectively. The DMSO control and 100 μg/mL concentrations indicated marginally higher proliferation activity compared to the control, while 10 and 1 μg/mL concentrations had lower proliferation compared to the control. However, there were no statistically significant differences (*p* > 0.05) between the cell counts of all the treatment concentrations when compared to the control (Fig. 5a). An increase in the number of cells was observed as the concentration increased from 1 to 500 μg/mL, and then a decrease from 500 to 1000 μg/mL. This concentration-dependent trend by which Malaysian propolis exerts its effect on the proliferation of fibroblast cells can be seen in Fig. 5b.

**Fig. 6:**
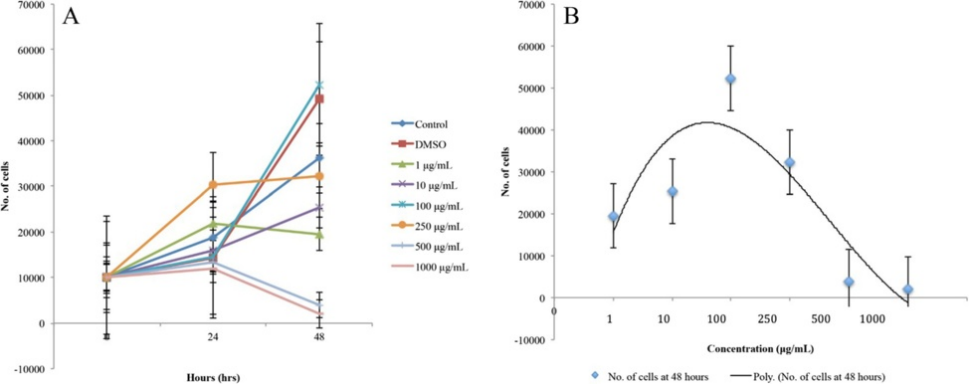
Proliferation of cells treated with Brazilian red propolis. **a** Line graph showing proliferation of treated and untreated cells using different concentrations of Brazilian red propolis at various time points. Values are expressed as mean number of cells ± standard error as indicated by the error bars. There was no significant difference between the mean number of cells of the control and treatments. **b** Polynomial trend line showing the concentration-dependent trend of the proliferation of cells treated with Brazilian red propolis at 48 h. Values are expressed as mean number of cells ± standard error as indicated by the error bars

Brazilian red propolis, the 100 μg/mL concentration showed the highest average cell count at 48 h (Fig. 6a). However, it only had a marginally higher number of viable cells when compared to the DMSO control. The 10 and 250 μg/mL concentrations indicated a slight increase in proliferation activity from 24 to 48 h, although this was still lower than that shown by the control. The 500 and 1000 μg/mL concentrations had a constant decrease in cell count throughout the entire duration. All the concentrations apart from the 100 μg/mL concentration resulted in less proliferation activity compared to the control. However, the differences between the mean number of cells proliferated in the presence of the treatments and the control were not statistically significant (*p* > 0.05). Nevertheless, an increasing trend between concentration and cell count was observed until 100 μg/mL, and then a decreasing trend was observed as the concentration increased (Fig. 6b).

## Discussion

Propolis has been used for many medicinal purposes due to its antimicrobial, anti-inflammatory and antioxidant properties [13]. These antimicrobial, antioxidant and anti-inflammatory activities are not distinct to the healing process, rather they facilitate it. In an infected wound, neutrophil cells increase in number and induce inflammation. It has been suggested that sustained inflammation may lead to marked necrosis and tissue damage, causing the healing process to be hampered [9]. Neutrophil cells as well as macrophages also generate oxygen free radicals that impede cellular regeneration. The antimicrobial property of propolis would rid the wound of unwanted organisms, thus consequently reducing the inflammation and the amount of free radicals produced [14]. Kilicoglu *et al.* [15] studied the effect of propolis on the healing of colon anastomosis in rats and observed that in the propolis group, fibroblast proliferation began soon after the number of neutrophil cells decreased. They also found that lymphocytes appeared earlier in that group compared to the control group. This suggests that propolis quickens the wound healing process by reducing acute inflammation and stimulating macrophage and T-lymphocyte activity.

The present study observed the effect of Malaysian and Brazilian red propolis on the proliferative phase of healing; specifically, the migration and proliferation of fibroblast cells, and the results were in accordance with previous studies [15, 16]. Kilicoglu *et al.* [15] observed that fibroblast proliferation, activation and synthesis capabilities were better in the presence of propolis than in its absence. Oliveira *et al.* [16] shared a similar view that propolis speeds up the healing process not only through its anti-inflammatory effect, but also by direct action on fibroblast proliferation.

Günay *et al.* [17] used propolis gel containing flavonoids and Caffeic acid phenylethyl ester (CAPE) to assess its effect on fibroplasia and epithelialization in post tooth extraction wounds and observed that although it had no significant action on fibroblast growth, it enhanced epithelialization. On the other hand, Borges *et al.* [18] reported an opposing observation on the effect of Tubi-bee propolis on glioblastoma and normal fibroblast cell lines. They found that propolis exerted a strong inhibition on the proliferation of both cell lines. Although not related to fibroblast cells, Meneghelli *et al.* [12] had a similar observation in that southern Brazilian autumnal propolis decreased cell viability, inhibited cell proliferation and migration, and capillary tube formation in human umbilical vein endothelial cells (HUVEC).

In order to evaluate the healing effect of Malaysian and Brazilian red propolis, migration and proliferation assays were carried out using normal human fibroblast cells. These assays were chosen as they are economical and commonly used in studying cell migration, proliferation and viability. In the present study, it was observed that the effect of both Malaysian and Brazilian red propolis on cell migration and proliferation followed a concentration-dependent optimum curve. With Malaysian propolis, the migration rates of fibroblast cells treated with 1 μg/mL up to 1000 μg/mL were faster compared to the control. The 100 μg/mL showed an anomalous result, as it did not coincide with the increasing trend in the healing effect of propolis from 1 μg/mL to 250 μg/mL. This could be due to an inconsistency in the initial seeding, as a higher cell density coincides with a decrease in the migration speed. This is evidenced by Lee *et al.* [19]. Abercrombie and Heaysman [20] also showed that the rate of cell movement is inversely proportional to the number of cells in contact with it. The highest overall migration rate was seen at 250 μg/mL, making this concentration the most optimum for wound healing. Although there have been no previous studies on Malaysian propolis, this finding is similar to that of an unpublished study that found propolis extract concentrations of 250, 300, 350, 400, 450 and 500 μg/mL resulted in significant proliferation of human periodontal ligament fibroblasts (HPDLFs) [21]. The rate of migration decreased after 250 μg/mL, showing that its efficacy declines at higher concentrations. This finding could be supported by the research done by Draganova-Filipova *et al. * [22] who compared the effects of propolis and CAPE on proliferation and apoptosis of McCoy-Plovdiv cell line and observed that high concentrations of propolis and CAPE caused apoptosis-induced cell death in McCoy-Plovdiv cells.

Brazilian red propolis, on the other hand, did not show much of a positive effect on wound healing. All the concentrations showed a slower rate of migration compared to the control, except for 10 μg/mL, which had an almost equal average migration rate to the control. This suggests that Brazilian red propolis may be toxic to cells above 10 μg/mL, as it inhibits their migration. The anomalous result using 1 μg/mL could be due to the insufficient duration of experiment as the migration rate could possibly increase after 24 h. After 10 μg/mL, the migration rate showed a decreasing trend as the concentrations increased. There has been no scientific study done on the effect of Brazilian red propolis on fibroblast cells. However, a study carried out by de Funari *et al*. [23] in 2007 on the effect of Brazilian green propolis on the viability of mouse fibroblasts showed that from 0.12 to 7.81 μg/mL, propolis revealed no statistically significant differences from the control, but concentrations of 31.25 μg/mL or more were toxic to the cells. This corroborates with the results of the present study, as 10 μg/mL is in between 7.81 and 31.25 μg/mL, and the concentrations above 10 μg/mL proved to be increasingly inhibitory to the migration of fibroblast cells, as they got higher.

For the proliferation assay using Malaysian propolis, the average number of cells increased as the concentrations increased, in a trend-like fashion, peaking at 500 μg/mL at 48 h, and then decreasing slightly with 1000 μg/mL. All the concentrations showed better proliferation activity compared to the control, except for 1 and 10 μg/mL. The low number of cells in the presence of these two concentrations indicates minimal growth activity, and this could be due to low initial seeding of cells. The difference in the optimum propolis concentrations for cell proliferation (500 μg/mL) and migration (250 μg/mL) as seen in the present study is in agreement with a study carried out by de Donatis *et al.* [24] that reported that high concentrations of platelet derived growth factor (PDGF) stimulated fibroblast cell proliferation, while low concentrations promoted cell migration. They observed that at low concentrations, the signaling pathways associated with cytoskeleton reorganization for cell movement are highly activated, whereas at high concentrations, the pathways associated with mitogenesis induction are stimulated. Hence, they speculate that the decision of the cell to migrate or proliferate depends on the various endocytic pathways that respond to different concentrations.

Taken together, the present study demonstrates that Malaysian and Brazilian red propolis have differing effects on fibroblast migration and proliferation. Malaysian propolis showed an overall positive effect on both assays compared to the control, and it followed a concentration-dependent curve with 250 μg/mL being the most optimum concentration for cell migration and 500 μg/mL for cell proliferation. Brazilian red propolis, on the other hand, showed only a slight increase in fibroblast migration and proliferation compared to the control at 10 and 100 μg/mL, respectively. It also followed a concentration-dependent curve, with concentrations above these points conferring inhibitory effects on both migration and proliferation. The difference in effects between Malaysian and Brazilian red propolis could be due to the difference between their chemical compositions.

Most propolis samples from around the world have been known to contain flavonoids [25]. Flavonoids from various sources have been found to be useful in wound healing. Geethalakshmi *et al.* [26] reported that the flavonoid fraction from *Sphaeranthus amaranthoides* highly increased the rate of wound contraction and epithelialization compared to silver sulphadiazine. A flavonoid derivative, quercetin 3-O-glucoside, from *Sambucus ebulus L.* leaves was also found to exert significant wound healing action [27]. Thus, it could be explained that flavonoids are responsible for the healing properties of Malaysian and Brazilian red propolis. However, the difference in their effects can be explained by a study done by Batista *et al.* [28], which showed that although Brazilian red propolis contained a higher concentration of total flavonoids than Brazilian green propolis, it was not as effective as green propolis in healing wounds. The reason for this could be the high concentration of isoflavonoids contained in Brazilian red propolis. Isoflavonoids have been found to exert anticancer activity – genistein, an isoflavone, inhibits the action of protein tyrosine kinase (PTK), topoisomerase II (has a role in DNA replication, transcription and repair) and matrix metalloprotein (MMP9), as well as down regulates VEGF, resulting in the suppression of cell growth and proliferation [29]. Therefore, the high concentration of isoflavonoids could account for the minimal wound healing effect and strong anti-proliferative property of Brazilian red propolis.

Propolis also increases expression of a number of genes that promote wound healing such as fibroblast growth factor 18 (FGF18) and vascular endothelial growth factor A (VEGFA). FGF18 is a pleiotropic growth factor that induces proliferation in various tissues. Hu *et al.* [30] observed that FGF18 caused a dose-dependent increase in the DNA synthesis of NIH3T3 fibroblast cell line. Through an MTS cell proliferation assay, they further established that FGF18 stimulates growth of fibroblast cells. Sonvilla *et al.* [31] reported that FGF18 induced the proliferation and migration of colon-associated fibroblast cells. The cell proliferation mechanism employed by FGFs is thought to be by a dual receptor system, which is through signal transducing FGF receptors (FGFR) and heparan sulphate (HS) proteoglycans [32]. A recent study by Miyaoka *et al.* [33] identified FGF18-dependent proliferation of Ba/F3 cells, that expressed FGFR3c, was facilitated by cysteine-rich FGF receptor (Cfr).

Vascular endothelial growth factor A (VEGFA), also known as VEGF, is a member of the PDGF/VEGF growth factor family and is most commonly associated with induction of endothelial cell growth and migration. However, studies have also shown that VEGF stimulates fibroblast proliferation [34–36]. In 2009, Li *et al.* [34] found that VEGF significantly promoted the proliferation of human and rabbit Tenon fibroblasts *in vitro*. Larsson-Callerfelt *et al.* [35] investigated the function of VEGF in fibroblast proliferation, migration and contractility using HFL-1 human lung fibroblasts. They observed that high concentrations of VEGF increased proliferation while low concentrations resulted in decreased migration rate. VEGF did not seem to affect fibroblast contractility. A study by Wu *et al.* [36] showed that endogenous VEGF stimulates fibroblast proliferation through glucocorticoid receptor (GR) pathway. A study by Khomenko *et al.* [37] found that it enhances synthesis of bFGF (Fibroblast Growth Factor), PDGF and VEGF. On the other hand, propolis down regulates follistatin (FST), a suppressor of cell proliferation. Yamashita *et al.* [38] found that FST strongly inhibited proliferation of NRK-49F rat kidney fibroblast cell line by antagonizing activin A. Activin A is a member of transforming growth factor- β (TGF- β) superfamily that regulates cell growth and differentiation [39].

Wound healing is a combination of various stages and is facilitated by antimicrobial, anti-inflammatory and anti-oxidant activities. Hence, to have an in-depth view of the effect of propolis on the entire wound healing process, further studies need to be done on the other stages of wound healing involving different cell types and various mechanisms. To further compare the effect of different propolis on wound healing, gene expression analysis needs to be carried out using different types of propolis at different concentrations at various time points.

### Limitation of the study

This study used an *in vitro* model and as such, this model may not fully reflect the wound healing situation which is a complex series of processes *in vivo*. Therefore, this work will be repeated using an animal model.

## Conclusion

Malaysian and Brazilian red propolis show potential to assist in wound healing, depending on their concentration.
